# TargetSA: adaptive simulated annealing for target-specific drug design

**DOI:** 10.1093/bioinformatics/btae730

**Published:** 2024-12-04

**Authors:** Zhe Xue, Chenwei Sun, Wenhao Zheng, Jiancheng Lv, Xianggen Liu

**Affiliations:** College of Computer Science, Sichuan University, Chengdu, Sichuan 610065, China; College of Computer Science, Sichuan University, Chengdu, Sichuan 610065, China; College of Computer Science, Sichuan University, Chengdu, Sichuan 610065, China; College of Computer Science, Sichuan University, Chengdu, Sichuan 610065, China; College of Computer Science, Sichuan University, Chengdu, Sichuan 610065, China; Laboratory of Anesthesia and Critical Care Medicine, Department of Anesthesiology, Translational Neuroscience Center, West China Hospital, Sichuan University, Chengdu, Sichuan 610041, China

## Abstract

**Motivation:**

The burgeoning field of target-specific drug design has attracted considerable attention, focusing on identifying compounds with high binding affinity toward specific target pockets. Nevertheless, existing target-specific deep generative models encounter notable challenges. Some models heavily rely on elaborate datasets and complicated training methodologies, while others neglect the multi-constraint optimization problem inherent in drug design, resulting in generated molecules with irrational structures or chemical properties.

**Results:**

To address these issues, we propose a novel framework (TargetSA) that leverages adaptive simulated annealing (SA) for target-specific molecular generation and multi-constraint optimization. The SA process explores the discrete structural space of molecules, progressively converging toward the optimal solution that fulfills the predefined objective. To propose novel compounds, we first predict promising editing positions based on historical experience, and then iteratively edit molecular graphs through four operations (insertion, replacement, deletion, and cyclization). Together, these operations collectively constitute a complete operation set, facilitating a thorough exploration of the drug-like space. Furthermore, we introduce a reversible sampling strategy to re-accept currently suboptimal solutions, greatly enhancing the generation quality. Empirical evaluations demonstrate that TargetSA achieves state-of-the-art performance in generating high-affinity molecules (average vina dock −9.09) while maintaining desirable chemical properties.

**Availability and implementation:**

https://github.com/XueZhe-Zachary/TargetSA

## 1 Introduction

Target-specific drug design involves developing molecules that interact with a disease-related target, aiming to enhance affinity and selectivity while minimizing off-target effects (Long *et al.* 2022, Wang *et al.* 2022). This methodology is crucial in modern drug discovery, as it facilitates the creation of more effective medications with fewer side effects compared to nonspecific drugs. Successful target-specific drug design relies on precise 3D structures of target proteins. Recent advancements in structural biology techniques (Ziegler *et al.* 2021) and the emergence of artificial intelligence tools like AlphaFold (Jumper *et al.* 2021) have enhanced the detailed resolution of human protein structures. These innovations significantly improve the prospects for target-specific drug design.

It is common to characterize target-specific drug design as a multi-constraint optimization problem (Fromer and Coley 2023, Qu *et al.* 2024). Optimization objectives typically encompass the binding affinity of protein–ligand complexes and chemical properties of ligands (drug-likeness, synthetic difficulty, solubility, toxicity, etc.). Among these objectives, docking affinity is particularly significant and is commonly evaluated through docking simulations (Alon *et al.* 2021).

The prevailing method employed in target-specific drug design is virtual screening (Gimeno *et al.* 2019, Liu *et al.* 2024), which utilizes computational modeling, intelligent search algorithms, and domain-specific expertise to systematically explore each molecule within a library and establish comprehensive rankings (Chenthamarakshan *et al.* 2020). However, the inherent discreteness and sparsity of molecular space make it computationally impractical to screen all drug-like molecules, which are estimated to number between 10^23^ and 10^60^ (Polishchuk *et al.* 2013).

Instead of screening a fixed library, generative methods offer a more promising avenue for exploring the vast chemical space. Deep generative models (DGMs) typically map molecules into a continuous latent space and are trained to capture the distribution of existing active compounds (Du *et al.* 2022). However, their effectiveness in generating molecules with desired properties is limited if the training data do not closely align with these properties (Zhou *et al.* 2024). Furthermore, the heavy reliance on extensive real-world training data and the intricacy of the training process can result in suboptimal optimization outcomes (Xie *et al.* 2021). An alternative approach, combinatorial optimization methods (COMs), directly navigates the discrete chemical space to pinpoint optimal potential drug candidates. Although COMs often surpass DGMs (Jensen 2019), their potential in target-specific drug design remains largely underexplored.

Prior research endeavors have leveraged simulated annealing (SA) for molecule generation (Liu *et al.* 2021, Hao *et al.* 2024). However, this method focuses solely on optimizing fundamental chemical properties like water solubility, without customizing molecules for specific target pockets. Moreover, the presence of “activity cliffs” suggests that even minor modifications to the chemical structure can precipitate unforeseeable and dramatic changes in properties (Stumpfe *et al.* 2019). Traditional SA solely considers the properties of the current molecule candidate and lacks a long-term perspective for molecular editing, potentially discarding compounds with future potential despite their temporary unfavorable attributes.

To overcome the aforementioned challenges, we propose an innovative framework employing an adaptive SA algorithm for target-specific molecular generation and multi-constraint optimization. This framework integrates metaheuristics, particularly SA, into powerful neural networks to restrict the search space of drug-like compounds. Firstly, we formulate a comprehensive objective function that encompasses the property profiles of the generated molecules (docking affinity, drug-likeness, and synthesis difficulty), along with essential constraints (molecular validity and similarity). Subsequently, we consider locally edited structures, achieved through operations including insertion, replacement, deletion, and cyclization, as analogous to neighborhoods of a given structure, thereby forming the sampling candidates for SA. At each step, TargetSA proposes potential edits by a candidate molecule generator and then accepts or rejects the proposal based on sample quality. Initially, the SA algorithm operates at a high temperature, increasing the likelihood of accepting inferior samples to escape local optima. As optimization progresses, the temperature gradually cools down, facilitating convergence toward an optimal solution.

Our main contributions can be summarized as follows:

1. A graph editing procedure is employed to generate novel molecules. Four straightforward yet effective editing operators constitute a complete operation set suitable for molecular generation from scratch and molecular optimization tasks (Section 2.5.3).
2. A history-guided position predictor is designed to identify promising editing positions in the molecule based on historical success experience, greatly enhancing generation quality compared to random selection (Section 2.5.1).
3. A reversible sampling strategy is introduced in SA to expand the exploration scope in chemical space and mitigate the impact of “activity cliffs” (Section 2.6).
4. We present the first SA framework for target-specific drug design, achieving SOTA performance in terms of docking-related metrics (Section 3.2).

## 2 Materials and methods

### 2.1 Related work

#### 2.1.1 Atom-based molecular generation

The atom-based molecular generation incrementally connects atoms to construct complete molecular structures, offering high flexibility for *de novo* drug design (Meyers *et al.* 2021). Many early models adopt SMILES/SMARTS strings as a text-based representation for atom-by-atom molecule generation. By pre-training on extensive molecular structures, the generative model can learn prior knowledge that encapsulates the grammar and syntax of valid SMILES (Jensen 2019). However, given that strings lack permutation invariance and structural information (Ragoza *et al.* 2022), graphs emerge as a more prevailing representation of molecules. Recent methodologies such as AR (Luo *et al.* 2021), Pocket2Mol (Peng *et al.* 2022), and GraphBP (Liu *et al.* 2022) utilize 3D graph neural networks to generate ligands atom-by-atom in an autoregressive manner.

#### 2.1.2 Fragment-based molecular generation

Fragment-based molecular generation methods use predefined scaffolds as fundamental building blocks to construct the entire molecule fragment by fragment. Compared to atom-based methods, this approach offers a reduced search space and facilitates precise control over the generation quality. For instance, JT-VAE generates a tree-shaped scaffold from chemical substructures and then integrates them into a complete molecule (Jin *et al.* 2018). Similarly, FLAG extracts high-frequency molecular fragments from a database and employs a 3D GNN to forecast the subsequent fragment for autoregressive assembly (Zhang *et al.* 2022). Building upon FLAG, DrugGPS incorporates structural details of subpockets and devises a global interaction graph to simulate the interactions between subpocket prototypes and molecular fragments (Zhang and Liu 2023).

#### 2.1.3 Diffusion-based molecular generation

Diffusion models establish a Markov chain of diffusion steps to slowly introduce random noise to data and subsequently learn to reverse the diffusion process to generate desired data samples from the noise. Recently, diffusion models have been widely adopted for target-specific drug design. Examples such as DiffSBDD (Schneuing *et al.* 2022) and TargetDiff (Guan *et al.* 2022) concentrate on learning the distribution of atom types and positions from a standard Gaussian prior based *via* the diffusion process. Additionally, DecompDiff (Guan *et al.* 2023) undertakes an exploration of the distinct roles of atoms within the ligand. DecompOpt (Zhou *et al.* 2024) introduces a controllable diffusion process for molecular optimization tasks. The above three generative paradigms share a common limitation, where subsequent generation quality highly depends on the state of the previous step.

#### 2.1.4 Editing-based molecular generation

In contrast to autoregressive and diffusion-based molecular generation approaches, molecular editing also plays a crucial role in drug design. For example, GA (Nigam *et al.* 2020) employs a genetic algorithm to edit 1D SELFIES strings, while MolDQN (Zhou *et al.* 2019) optimizes molecular properties by adding or removing atoms and bonds. Approaches like MARS (Xie *et al.* 2021) and MIMOSA (Fu *et al.* 2021) focus on editing 2D molecules through fragment addition, deletion, or replacement, whereas MolEdit3D (Yang *et al.* 2024) adds or deletes rigid fragments in 3D space. In this work, we extend editing-based molecular optimization and generation for target-specific drug design. Specifically, we propose a step-wise molecular editing approach using four simple yet effective operations: insertion, replacement, deletion, and cyclization.

### 2.2 Problem definition

**Figure 1.**
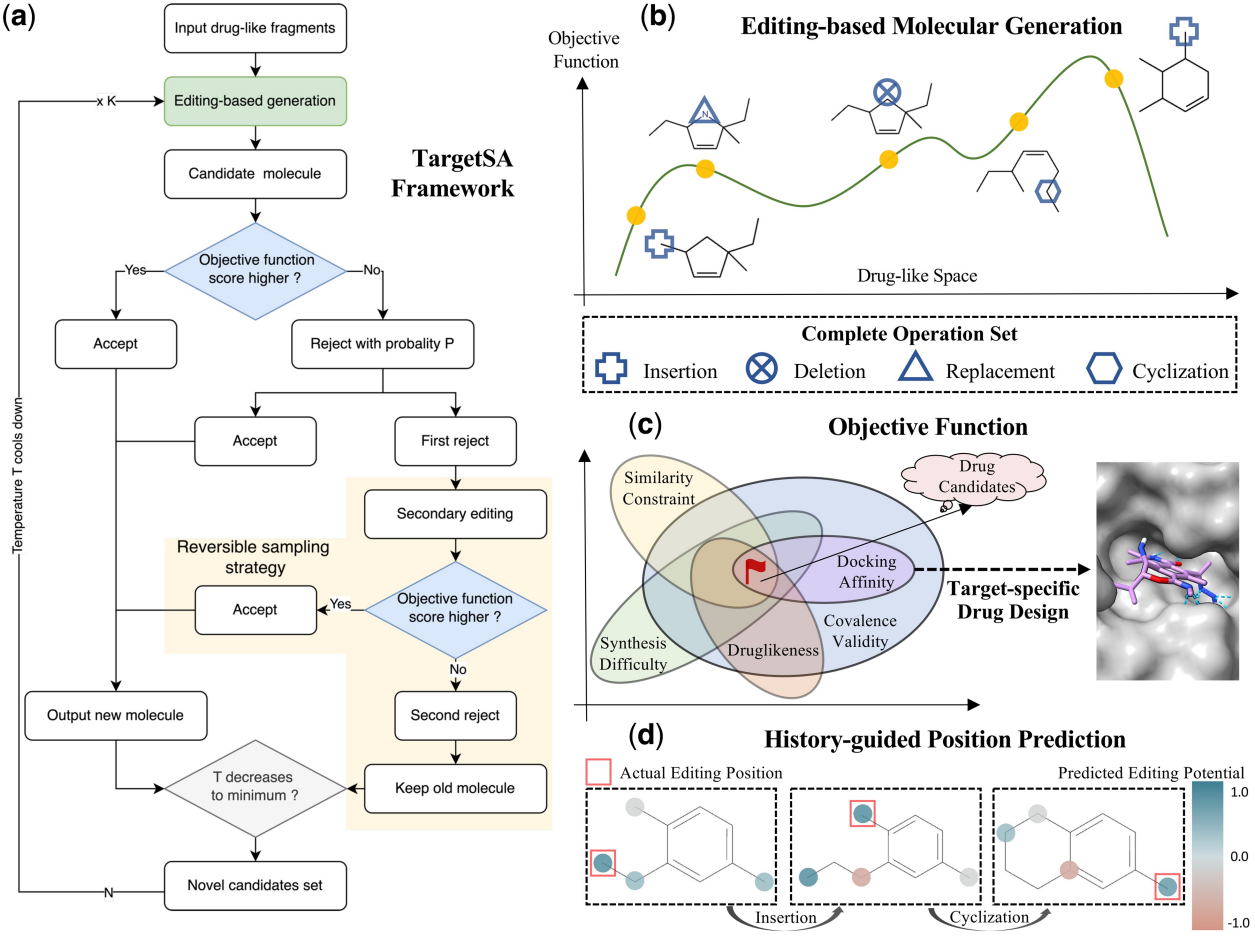
(a) TargetSA framework comprises the simulated annealing process and a reversible sampling strategy. (b) We generate molecules using a complete set of four graph editing operations, systematically exploring the drug-like chemical space to identify novel compounds with high objective function scores. (c) A sophisticated objective function is formulated for multi-constraint molecular optimization, incorporating docking simulations tailored to target-specific drug design. (d) A history-guided position predictor forecasts the editing potential of each atom within a molecule.

In this section, we introduce our framework, TargetSA, as illustrated in Fig. 1. We first provide a detailed description of the SA process and the associated objective function. Following this, we present an editing-based molecular candidate generator, coupled with a history-guided position predictor for constrained molecular generation and optimization. Finally, we propose a reversible sampling strategy designed to re-accept currently suboptimal molecules, greatly enhancing the quality of generation results.

####  

Let X denote the large search space of molecules, and 2D molecular graph sample at step *t* be $x_{t}=\{V_{t},E_{t}\}$, where $V_{t}=(v_{t,1},\ldots ,v_{t,k},\ldots ,v_{t,n})$ is the set of nodes, *E_t_* is the set of edges, and *n* is the number of nodes. The objective function f(x) is predefined to ensure that generated molecules satisfy specific chemical properties (e.g. Docking Affinity, QED, SA, etc.). During the optimization process, TargetSA proposes new molecular candidates by iteratively editing molecular graphs, aiming to search for molecules x that **maximize**  f(x).

### 2.3 SA process

SA is a stochastic optimization algorithm that explores discrete chemical space to achieve a predefined objective through local modifications. At each iteration, SA selects a new molecular candidate and determines its acceptance based on a probability that considers both solution quality and the current annealing temperature. The acceptance probability remains at one for better solutions, gradually decreasing to zero for worse ones. Theoretically, SA is guaranteed to converge to the global optimum in a finite problem, provided the proposal mechanism and temperature schedule meet certain conditions (Granville *et al.* 1994).

During the SA process, at each step *t*, TargetSA edits the current molecule $x_{t}$ to propose a new candidate $x_{*}$. If candidate $x_{*}$ scores higher by f(x), i.e. $f(x_{*})>f(x_{t})$, it is accepted immediately. Otherwise, TargetSA tends to reject the proposal $x_{*}$, but may still accept it with a small probability $e^{\frac{f(x_{*})-f(x_{t})}{T}}$. In other words, the accepting probability is


(1) $$p(\text{accept}|x_{*},x_{t},T)=\min\left(1,e^{\frac{f(x_{*})-f(x_{t})}{T}}\right)$$

(2) $$T=\max\left\{T_{\text{min}},\frac{T_{\text{init}}}{t+1}\right\}$$

where *T* is the current temperature, $T_{\text{init}}$ represents the initial temperature, $T_{\text{min}}$ represents the final temperature, and t is the current iteration. If candidate $x_{*}$ is accepted, it becomes the input for the next step, $x_{t+1}=x_{*}$; If rejected, $x_{t+1}=x_{t}$.

At the beginning, the temperature T is set to a high value, even if $x_{*}$ is worse than $x_{t}$, there is still a high acceptance probability. This initial high temperature setting enables a thorough exploration of the chemical space. Subsequently, the temperature gradually decreases, facilitating convergence toward a specific optimal solution. The iterative SA process continues until the current T decreases to $T_{\min}$, resulting in a collection of novel compounds with superior objective function values compared to the original input $x_{0}$.

### 2.4 Objective function

We aim to identify novel compounds that bind tightly to the target and possess high drug-likeness and synthesis accessibility. Therefore, we need to optimize three properties:


(3) $$f_{p}(x_{*},x_{0})=\alpha f_{\text{Dock}}(x_{*})+\beta f_{\text{syn}}(x_{*})+\gamma f_{\text{QED}}(x_{*})$$

where $f_{\text{Dock}}(x_{*})$ indicates the docking affinity between the generated molecule and the target, calculated by docking software. $f_{\text{syn}}(x_{*})$ is the synthetic accessibility and $f_{\text{QED}}(x_{*})$ is the drug-likeness, calculated by RDKit. Settings of hyperparameters are in the Supplementary Material.

We have selected 112 drug-like fragments as the starting point for optimization, rather than generating from scratch. To promote structural diversity and avoid over-reliance on any single scaffold type, the Tanimoto similarity between fragments was constrained to remain below 0.8. Further screening criteria and visualizations of the selected drug-like fragments are in the Supplementary Material. Newly generated molecules are expected to exhibit novelty and diversity while preserving the structural characteristics of the input fragments. The similarity between the new molecule and input fragments should be controlled within a reasonable range. Furthermore, molecules that violate chemical rules must be excluded. Consequently, we design an additional function to enforce constraints on similarity and valency conditions:


(4) $$\text{Validity}(x_{*},x_{0})=f_{g}(x_{*})\cdot 1_{\left\{f_{\mathrm{sim}}(x_{*},x_{0})>\delta \right\}},$$

where *f_g_* indicates whether the sample $x_{*}$ is subject to the valency conditions or not, $f_{\text{sim}}$ measures the Morgan similarity between candidate $x_{*}$ and the input start molecule $x_{0}$. $1_{\left\{\cdot \right\}}$ is an indicator function that yields 1 when its argument is true, and 0 otherwise. In other words, a newly generated molecule $x_{*}$ is regarded as valid only if its grammar is correct and the similarity is larger than *δ*.

Finally, we combine the above two functions to define a sophisticated objective function of TargetSA. Notably, we assign negative infinity to the invalid molecules to prevent them from being sampled


(5) $$f(x_{*})=\{\begin{array}{cccc} & f_{p}(x_{*},x_{0}), & & \text{if}\;\text{Validity}(x_{*},x_{0})>0,\\ & -\infty , & & \text{if}\;\text{Validity}(x_{*},x_{0})\leq 0, \end{array}$$

### 2.5 Candidate molecule generator

We model the molecule generation task as a graph editing process. At each search step, the history-guided position predictor provides a potential editing position, and then the candidate molecule generator proposes new candidates through local editing operations sampled from a complete operation set.

#### 2.5.1 History-guided position predictor

Before modifying the molecular structure, selecting appropriate positions for editing is essential. Although random selection seems straightforward, some positions may not be conducive to effective edits. To address this, we design a position predictor based on historical experience. In short, we collected molecules with randomly selected positions and constructed matrixes that track the editing frequency of each atom, which serves as an indicator of its editing potential. We formulated the position prediction task as a node classification problem, leveraging a graph neural network to generate atom-level representations based on the molecular graph features.

To collect historical data, we execute the SA process under a random selection setting. Let X represent a set of accepted molecules, $X=(x_{0},\ldots ,x_{r},\ldots ,x_{m})$. $L=(l_{0},\ldots ,l_{r},\ldots ,l_{m})$ is the set of corresponding editing positions. We sample a molecule $x_{r}$ and its associated position $l_{r}$ by $x_{r}∼X,l_{r}∼L$.

Then, we construct a matrix to record the editing frequency of each atom in a molecule. Let *Q_r_* be the atomic editing frequency matrix for molecule $x_{r}$, and $q_{r,i}$ represents the *i*th row of matrix *Q_r_*, containing the editing frequency for the *i*th atom in molecule $x_{r}$. In other words, $Q_{r}={\left[q_{r,0},\ldots ,q_{r,n}\right]}^{T}$, and *n* represents the atom number of $x_{r}$. The atomic frequency matrix of the current molecule is calculated from the previous molecule’s matrix. At each step, the editing frequency of the selected position, along with its neighbor nodes, is incremented by one. If $x_{r+1}$ is generated from $x_{r}$ by editing at position *s*, $Q_{r+1}={\left[q_{r+1,0},\ldots ,q_{r+1,m}\right]}^{T}$ can be updated as


(6) $$q_{r+1,u}=\{\begin{array}{cccc} & q_{r,u}+1 & & \text{if}\;v_{r,u}\in N(v_{r,s})\cup v_{r,s},\\ & q_{r,u} & & \text{if}\;v_{r,u}\notin N(v_{r,s})\cup v_{r,s}, \end{array}$$

where $N(v_{r,s})$ denotes the set of neighboring nodes of node $v_{r,s}$, *m* is the atom number of $x_{r+1}$, and u∈[0,m].

Following the above preparation, we formulate the position prediction task as a node classification problem. We aim to learn a representation vector $h_{v_{r,i}}$ for node $v_{r,i}$, which facilitates the prediction of atom editing frequency. Our framework utilizes the graph isomorphism network (GIN) (Xu *et al.* 2018) to generate node representations for each atom, incorporating molecular graph features. These features include atom attributes like type and chirality, along with edge attributes such as bond types and stereochemistry. Details of the training procedure are in the Supplementary Material.

For a given molecule $x_{t}$, we use GIN to predict its editing frequency matrix $\hat{Q_{t}}$, and then select the top-*K* positions as a candidate set $L_{t}$. The final editing position $l_{t}$ is subsequently sampled from this set by Eq. (7). For the sake of conciseness, we define the aforementioned editing position prediction process as Eq. (8)


(7) $${\hat{Q}}_{t}=\text{GIN}(x_{t}),\;L_{t}=\text{top}-K_{{\hat{Q}}_{t}},\;l_{t}∼L_{t}$$

(8) l=Pos(x)

#### 2.5.2 Editing-based molecular generation

The following part elaborates on how to generate new molecules through editing operations. Take editing molecule $x_{t}$ at the position $l_{t}$ as an illustrative example. The insertion operator proposes a candidate node $v_{*}$ and a new edge $e_{\left(l_{t},*\right)}$ to connect the newly formed $v_{*}$ with $v_{t,l_{t}}$, given by


(9) $$x_{*}=\text{Insertion}(x_{t},l_{t},v_{t,l_{t}},e_{\left(l_{t},*\right)}),\;x_{*}=\{V_{*},E_{*}\}$$

(10) $$V_{*}=V_{t}\cup \{v_{t,l_{t}}\},\;E_{*}=E_{t}\cup e_{\left(l_{t},*\right)}$$

The deletion operator removes the atom at the selected position and its attached chemical bonds, so $x_{*}=\{V_{t}\setminus \{v_{t,l_{t}}\},E_{t}\setminus E_{l_{t}}\}$, where $E_{l_{t}}$ refers to a set of bonds connected to the atom $v_{t,l_{t}}$. Notably, removing the atom $v_{t,l_{t}}$ may break the molecule $x_{t}$ into a set of *n* substructures *S_t_* when $v_{t,l_{t}}$ is a linking atom. We check the validity of these substructures and then sample one as a candidate $x_{*}$.


(11) $$S_{t}=\text{Deletion}(x_{t},l_{t})=\{s_{1},\ldots ,s_{n}\},\;x_{*}∼S_{t}$$

For the replacement operator, it suggests a candidate node $v_{*}$ and the resulting candidate sample becomes $x_{*}=\{V_{*}=(v_{t,1},\ldots ,v_{t,l_{t}-1},v_{*},\ldots ,v_{t,n}),E_{t}\}$. Similarly, the cyclization operator links two adjacent atoms to establish a new bond, thereby creating a new ring structure.

Generally, let us define our editing operation set as A= {**insertion**, **replacement**, **deletion**, **cyclization**}. At step *t*, we sample an editing action $a_{t}$ from this set and then modify the input molecule $x_{t}$ at position $l_{t}$, given by


(12) $$a_{t}∼A,\;x_{*}=\text{Gen}(x_{0})=\text{Editing}(x_{t},l_{t},a_{t})$$

where $\text{Gen}(x_{0})$ means after a whole *t*-step editing process, the initial input $x_{0}$ is converted to $x_{*}$. Meanwhile, $\text{Editing}(x_{t},l_{t},a_{t})$ represent the current editing step *t*. The probabilities for each editing operation in the set A are explained in the Supplementary Material.

#### 2.5.3 Complete operation set

We define A={insertion,replacement,deletion,cyclization} as a complete operation set, which theoretically enables the sampling of every molecule within the drug-like space.

Definition. If an arbitrary molecule can be generated by applying a finite sequence of operations from the set S, then the operation set S is considered complete.

Theorem 1. *The above operation set is complete for molecule generation from scratch.*

Every molecule can be decomposed into tree structures and ring structures. A tree structure denotes a cycle-free substructure composed of a single atom or side chains, while ring structures encompass ring patterns like five-membered rings and benzene rings. Each tree structure can be derived from a single atom through insertion and replacement operators, while ring structures can be generated from tree structures or other ring structures via the cyclization operator. Therefore, it can be asserted that set A is complete for generating molecules from scratch. Details of the proof are provided in the Supplementary Material.

Theorem 2. *The above operation set is complete for molecule optimization.*

The molecular optimization process involves transforming molecule $x_{a}$ into $x_{b}$. One pathway is editing $x_{a}$ by iteratively deleting components until reaching the maximum common substructure $x_{c}$ shared between $x_{a}$ and $x_{b}$. Subsequently, molecule $x_{b}$ can be generated from $x_{c}$.


(13) $$x_{c}=x_{a}\cap x_{b}$$

(14) $$x_{c}=\text{Gen}(x_{a}),\;a_{c}∼A_{c},\;A_{c}=\{\mathrm{deletion}\}$$

(15) $$x_{b}=\text{Gen}(x_{c}),\;a_{b}∼A_{b},$$

$$A_{b}=\{\mathrm{insertion},\mathrm{replacement},\mathrm{cyclization}\}$$

since $A=A_{c}\cup A_{b}$, it is safe to say that A is complete for molecule optimization.

### 2.6 Reversible sampling strategy in SA

The traditional SA algorithm typically rejects candidates $x_{*}$ with lower objective function scores (Hwang 1988). However, our reversible sampling strategy permits $x_{*}$ to undergo a secondary editing process before final rejection, given by


(16) $$a_{t}^{\prime }∼A,\;l_{t}^{\prime }=\text{Pos}(x_{*}),\;x\prime_{*}=\text{Editing}(x_{*},l_{t}^{\prime },a_{t}^{\prime })$$

where $l_{t}^{\prime }$ represents the predicted editing position of candidate $x_{*}$. If the resulting molecule $x\prime_{*}$ from this secondary editing process remains unsatisfactory, i.e. $f(x\prime_{*})<f(x_{t})$, candidate $x_{*}$ is truly discarded. Otherwise, $x_{*}$ can be accepted since it exhibits potential for modification to a better candidate $x\prime_{*}$. This re-accepting process is defined as “**reversible sampling**”. Our strategy broadens the search within the chemical space by temporarily accepting suboptimal candidates, demonstrating its efficacy in experimental results. Algorithm 1 presents a comprehensive depiction of the TargetSA framework.

**Algorithm 1.** The TargetSA framework

**Require:** Initial sample $x_{0}$

1: **for**  t∈{0,…,N}  **do**

2:  $T=\max\left\{T_{\text{min}},\frac{T_{\text{init}}}{t+1}\right\}$

3:  Select an editing position $l_{t}=\text{Pos}(x_{t})$ by Eqn. (8) and an editing operation $a_{t}∼A$ by Eqn. (12)

4:  Edit the current sample $x_{t}$ and propose a candidate $x_{*}=\text{Editing}(x_{t},l_{t},a_{t})$ by Eqn. (12)

5:  Compute the accepting probability $p_{\text{accept}}$ by Eqn. (1)

6:  With probability $p_{\text{accept}},\;x_{t+1}=x_{*}$

7:  With probability $1-p_{\text{accept}}$, use $x_{*}$ as the input molecule for the reversible sampling strategy and sample a new candidate $x\prime_{*}$ by Eqn. (16)

8:  **if**  $f(x\prime_{*})>f(x_{t})$  **then**  $x_{t+1}=x_{*}$; **else**  $x_{t+1}=x_{t}$

9: **end for**

10: **return**  $x_{\tau }$ s.t. $\tau =\arg\max_{\tau \in \{1,\ldots ,N\}}f(x_{\tau })$

## 3 Results and Discussion

### 3.1 Experimental setup

#### 3.1.1 Dataset

We evaluated the performance of our framework on the Crossdocked2020 dataset (Francoeur *et al.* 2020), comprising a training set of 100 000 protein–ligand complex pairs and a test set of 100 pairs, filtered through a process similar to previous studies (Luo *et al.* 2021, Guan *et al.* 2022).

#### 3.1.2 Baselines

Baselines can be categorized into four types. (1) variational autoencoder: LiGAN (Ragoza *et al.* 2022); (2) autoregressive models: AR (Luo *et al.* 2021), Pocket2Mol (Peng *et al.* 2022), GraphBP (Liu *et al.* 2022), FLAG (Zhang *et al.* 2022), DrugGPS (Zhang and Liu 2023); (3) diffusion models: TargetDiff (Guan *et al.* 2022), DecompDiff (Guan *et al.* 2023), IPDiff (Huang *et al.* 2023), DecompOpt (Zhou *et al.* 2024), BindDM (Huang *et al.* 2024); (4) genetic algorithm: RGA (Fu *et al.* 2022).

#### 3.1.3 Metrics

We utilized established binding-related and property-related metrics from recent works (Guan *et al.* 2022, Peng *et al.* 2022) to estimate generation quality. (1) **Vina Dock**: the binding affinity between generated molecules and target pockets, calculated by AutoDock Vina; (2) **High affinity**: the percentage of generated molecules with docking affinities higher than the given reference; (3) drug-likeness (**QED**); (4) synthesizability (**SA**); (5) diversity (**Div.**): average pairwise dissimilarity of generated molecules for a binding pocket; (5) Lipinski’s rule of five (**Lip.**) assesses how many of its rules a molecule meets, offering an empirical assessment of drug-likeness (Lipinski *et al.* 2012).

### 3.2 Generation results

**Figure 2.**
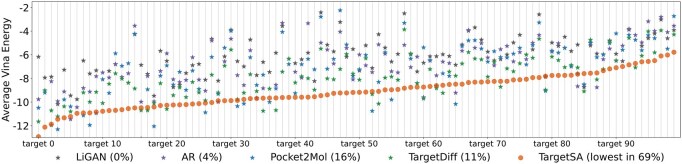
Average Vina energy for different models (AR, Pocket2Mol, TargetDiff, and TargetSA) across 100 testing binding targets. The target numbers are arranged based on the average docking scores of molecules generated by TargetSA.

Figure 2 shows the average docking scores of molecules generated across 100 test pockets (lower is better). TargetSA achieves the highest binding affinity in 69% of targets, while LiGAN, AR, Pocket2Mol, and TargetDiff achieve the best binding in 0%, 4%, 16%, and 11% of cases, respectively.

Table 1 summarizes the properties of generated molecules. For binding-related metrics, TargetSA outperformed all baselines. Specifically, 79.4% of generated molecules exhibited tighter binding to the target compared to the reference.

**Table 1.** Property summary of reference molecules and generated molecules by different models.

Model Metric	Vina dock (↓)		High affinity (↑)		QED (↑)		SA (↑)		Div. (↑)		Lip. (↑)
	Avg.	Med.	Avg.	Med.	Avg.	Med.	Avg.	Med.	Avg.	Med.	Avg.
LiGAN	−6.33	−6.20	21.1%	11.1%	0.39	0.39	0.59	0.57	0.66	0.67	4.05
GraphBP	−4.80	−4.70	14.2%	6.7%	0.43	0.45	0.49	0.48	**0.79**	**0.78**	4.45
AR	−6.75	−6.62	37.9%	31.0%	0.51	0.50	0.63	0.63	0.70	0.70	4.79
Pocket2Mol	−7.15	−6.79	48.4%	51.0%	0.56	0.57	**0.74**	**0.75**	0.69	0.71	4.89
FLAG	−6.96	−7.02	44.5%	48.6%	0.55	0.56	**0.74**	0.74	0.70	0.69	4.87
DrugGPS	−7.27	−7.35	56.5%	59.0%	**0.62**	**0.62**	**0.74**	0.73	0.68	0.69	**4.92**
TargetDiff	−7.80	−7.91	58.1%	59.1%	0.48	0.48	0.58	0.58	0.72	0.71	4.49
DecompDiff	−8.39	−8.43	64.4%	71.0%	0.45	0.43	0.61	0.60	0.68	0.68	4.40
IPDiff	−8.57	−8.51	69.5%	75.5%	0.52	0.53	0.61	0.59	0.74	0.73	–
BindDM	−8.41	−8.37	64.8%	71.6%	0.51	0.52	0.58	0.58	0.75	0.74	–
DecompOpt	−8.98	−9.01	73.5%	93.3%	0.48	0.45	0.65	0.65	0.60	0.61	–
RGA	−8.01	−8.17	64.6%	89.3%	0.57	0.57	0.71	0.73	0.41	0.41	4.78
Reference	−7.45	−7.26	–	–	0.48	0.47	0.73	0.74	–	–	4.25
TargetSA-wo-pos	−8.30	−8.42	69.4%	86.2%	0.60	**0.62**	0.63	0.62	0.60	0.59	4.90
TargetSA	**−9.09**	**−9.18**	**79.4%**	**93.8%**	0.54	0.54	0.62	0.62	0.59	0.58	4.83

Arrows (↑, ↓) indicate the direction of better performance. For each metric, the best method is bolded.

As for QED values, TargetSA-wo-pos (TargetSA without the history-guided position predictor) greatly outperformed the reference and surpassed all baselines. Although TargetSA may not achieve the highest QED and SA scores, this outcome reflects a deliberate tradeoff between relatively lower property-related scores and higher binding affinity. This tradeoff can be attributed to two main factors: First, the docking score is weighted more heavily in our objective function, which leads TargetSA to prioritize docking affinity, sometimes at the expense of certain chemical properties. Second, as the optimization progresses, the generated molecules often increase in atomic number and form more ring structures, resulting in more complex structures and greater synthetic difficulty. This tradeoff has also been reported by Huang *et al.* (2024) and DecompDiff (Guan *et al.* 2023). It is important to recognize that in real-world drug discovery, QED and SA scores are typically used as preliminary filters, deemed acceptable within a reasonable range (Guan *et al.* 2022). Overall, TargetSA achieves a more nuanced balance between superior docking scores and reasonable chemical properties compared to baselines.

Regarding Div., molecules designed for a specific protein pocket should not exhibit excessive diversity, as each pocket has distinct structural characteristics that constrain molecular structures (Peng *et al.* 2022). Evaluation results of novelty and uniqueness (in Supplementary Material) show that TargetSA effectively generates molecules tailored to specific pockets while maintaining a balance of high diversity and novelty.

**Figure 3.**
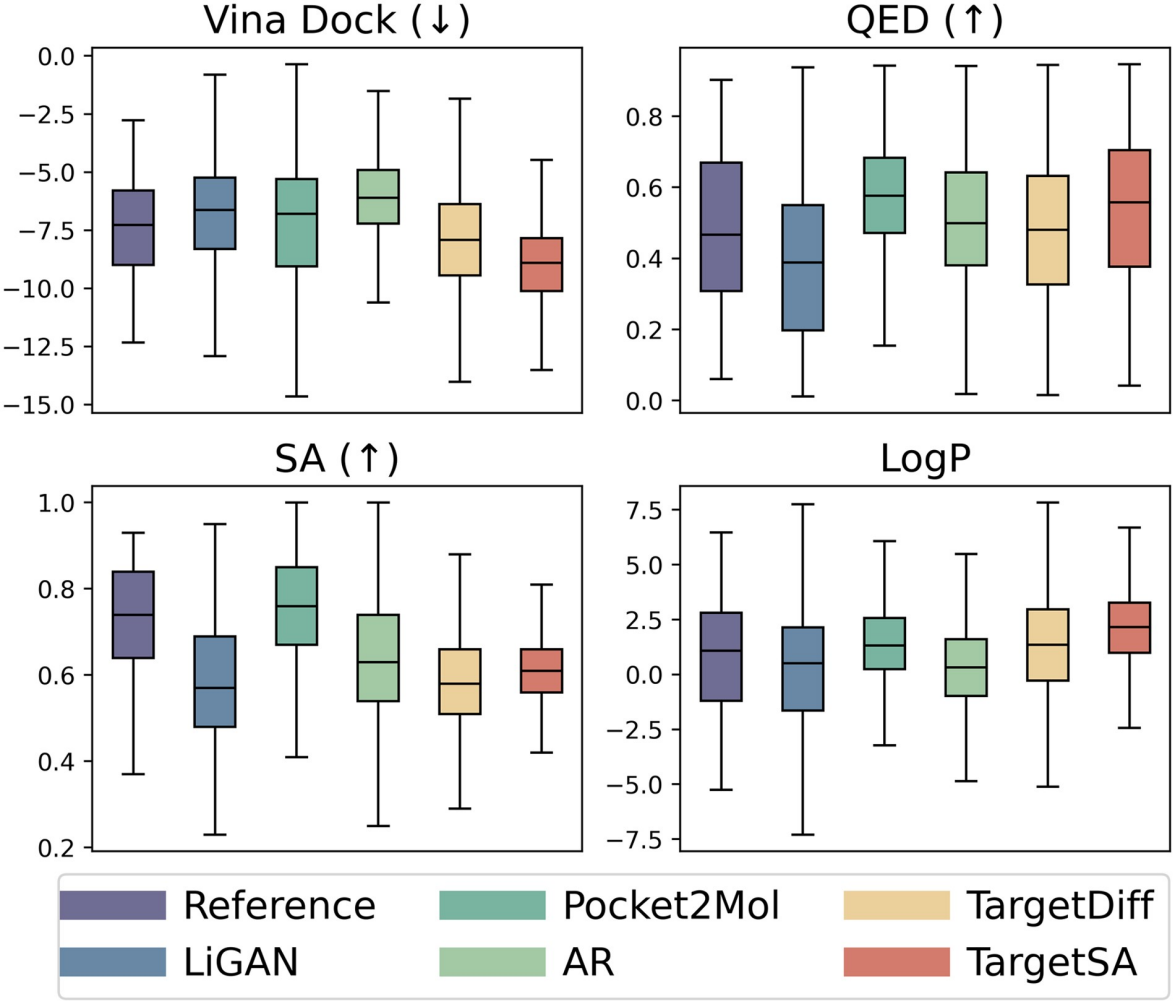
Distributions of binding affinity and chemical properties of molecules generated by different models.

Figure 3 further illustrates the distribution of chemical properties, emphasizing the high quality of generation results. Moreover, we compared TargetSA with previous editing-based methods, including MIMOSA (Fu *et al.* 2021) and MARS (Xie *et al.* 2021). Results in the Supplementary Material demonstrate that molecules generated by TargetSA still exhibit superior properties.

We must acknowledge that our algorithm is relatively time-consuming compared to other models, primarily for two reasons: first, the use of time-intensive docking simulations to guide the optimization process; second, the use of combinatorial optimization methods (e.g. SA), which typically sacrifice computation time to search for a global optimum. However, considering that real-world drug discovery can take decades, our runtime is comparatively negligible. Consequently, the slightly extended runtime does not hinder the practical applicability of TargetSA. Experimental results of time complexity are in the Supplementary Material. Moreover, we found that GPU-accelerated docking tools can significantly reduce computational runtime while maintaining comparable accuracy. Our future work will focus on further improving runtime efficiency by exploring GPU-based docking tools or leveraging neural networks for binding affinity prediction.

### 3.3 Ablation studies

In Table 2, we present a comprehensive evaluation of the contributions of each designed component. The full framework is denoted as TargetSA, while TargetSA-wo-pos represents the variant excluding the history-guided position predictor (randomly selecting positions), and TargetSA-wo-reverse corresponds to the version without the reversible sampling strategy. TargetSA-origin, in turn, lacks both components. The results indicate that both designed components significantly enhance binding affinity. Furthermore, TargetSA-origin outperforms the reference, underscoring the efficacy of our editing-based generation and optimization strategy.

**Table 2.** Generation results for different ablation versions.

Version	Avg. Vina dock	Med. Vina dock
TargetSA	−9.09	−9.18
TargetSA-wo-pos	−8.30	−8.42
TargetSA-wo-reverse	−8.58	−8.47
TargetSA-origin	−8.17	−8.21
Reference	−7.45	−7.26

### 3.4 Results on the binding MOAD dataset

To further showcase TargetSA’s proficiency, we evaluated TargetSA on the binding MOAD dataset (Hu *et al.* 2005), and we selected 130 pairs for testing followed by Schneuing *et al.* (2022). Notably, we utilized QVina to simulate the docking process and added two new baselines. **DiffSBDD** (Schneuing *et al.* 2022) is an SE(3)-equivariant 3D-conditional diffusion model. **KGDiff** (Qian *et al.* 2023) employs vina scores to guide the model, integrating domain knowledge of binding affinity into the denoising process of the diffusion model.

Table 3 illustrates that TargetSA produces ligands with notably superior binding affinities compared to baselines while demonstrating competitiveness in chemical metrics.

**Table 3.** Results on the 130 test pockets of Binding MOAD dataset.

Model Metric	Vina dock	High affinity	QED	SA	Lip.	Div.
DiffSBDD	−6.23	0.13	0.53	0.32	4.85	**0.72**
Pocket2Mol	−7.69	0.36	0.60	0.33	4.75	**0.72**
FLAG	−7.72	0.36	0.61	0.32	4.76	0.70
DrugGPS	−7.62	0.52	**0.64**	0.38	**4.86**	0.71
KGDiff	−7.82	0.66	0.59	0.54	4.79	0.61
TargetSA	**−7.95**	**0.69**	0.57	**0.62**	4.84	0.60
Reference	−6.83	–	0.60	0.34	4.84	–

For each metric, the best method is bolded.

### 3.5 Case studies in real-world drug discovery scenarios

To demonstrate the generalizability of our framework, we applied TargetSA to a real-world drug development scenario, selecting the functionally complex sigma-2 receptor as the target (PDB ID: 7M95). Recent studies have shown that sigma-2 ligands exhibit anti-allodynic properties in models of neuropathic pain (Alon *et al.* 2021), indicating that designing selective ligands for the sigma-2 receptor could potentially lead to the discovery of novel analgesic drugs.

**Figure 4.**
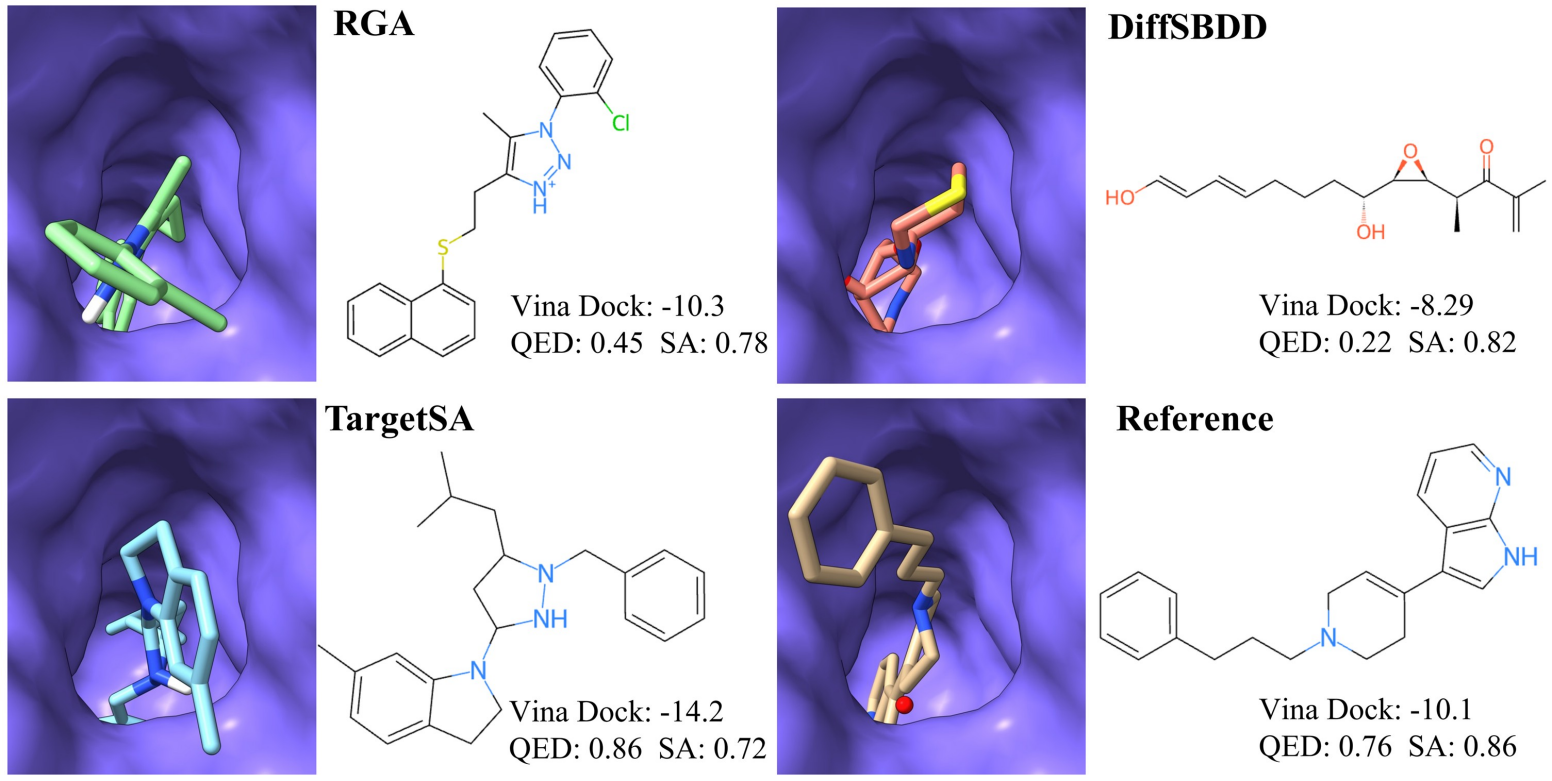
Results of different models for generating selective ligands against the sigma-2 receptor (PDB ID: 7M95).

As illustrated in Fig. 4, RGA (Fu *et al.* 2022) generated a complex structure that is not well-suited for the target pocket. Similarly, DiffSBDD (Schneuing *et al.* 2022) produced a long-chain molecule that failed to capture the cavity effectively and had a low QED score. In contrast, the ligand generated by TargetSA fully occupied the pocket, greatly enhancing binding affinity while maintaining high QED and SA scores.

In brief, TargetSA can generate novel ligands for any target, since our method neither requires pre-training on extensive protein–ligand complexes nor retraining from scratch when the target changes. We also evaluate TargetSA’s performance on complex targets with long amino acid sequences (see Results in the Supplementary Material), demonstrating TargetSA’s great potential for broad application in real-world target-specific drug discovery scenarios.

## 4 Conclusion

To the best of our knowledge, TargetSA presents the first SA-based framework specifically designed for target-specific molecular generation and multi-constraint optimization. By integrating metaheuristics with powerful neural networks, we effectively constrain the search space of drug-like compounds. Our approach generates novel compounds by iteratively editing molecular graphs through four simple yet effective operations. The traditional search algorithm is revitalized in the new application scenario, achieving state-of-the-art performance in binding-related metrics compared with the latest deep generative models.

## Supplementary Material

btae730_Supplementary_Data

## Code availability

Our code is available at https://github.com/XueZhe-Zachary/TargetSA.

## References

[btae730-B1] Alon A , Lyu J, Braz JM et al Structures of the *σ*2 receptor enable docking for bioactive ligand discovery. Nature 2021;600:759–64. 34880501 10.1038/s41586-021-04175-x PMC8867396

[btae730-B2] Chenthamarakshan V , Das P, Hoffman S et al CogMol: target-specific and selective drug design for covid-19 using deep generative models. Adv Neural Inf Process Syst 2020;33:4320–32.

[btae730-B3] Du Y , Fu T, Sun J et al MolGenSurvey: a systematic survey in machine learning models for molecule design. arXiv, arXiv:2203.14500, 2022, preprint: not peer reviewed.

[btae730-B4] Francoeur PG , Masuda T, Sunseri J et al Three-dimensional convolutional neural networks and a cross-docked data set for structure-based drug design. J Chem Inf Model 2020;60:4200–15. 32865404 10.1021/acs.jcim.0c00411 PMC8902699

[btae730-B5] Fromer JC , Coley CW. Computer-aided multi-objective optimization in small molecule discovery. Patterns 2023;4:100678. 36873904 10.1016/j.patter.2023.100678 PMC9982302

[btae730-B600] Fu T, Xiao CAO, Li X et al MIMOSA: Multi constraint molecule sampling for molecule optimization. In: *Proceedings of the 35th AAAI Conference on Artificial Intelligence*, pp. 125–33. Palo Alto, CA: AAAI Press, 2021

[btae730-B6] Fu T , Xiao CAO, Li X et al MIMOSA: Multi-constraint molecule sampling for molecule optimization. In *Proceedings of the 35th AAAI Conference on Artificial Intelligence,* 2021; pp. 125-133. AAAI Press, Palo Alto, CA.

[btae730-B7] Fu T , Gao W, Coley C et al Reinforced genetic algorithm for structure-based drug design. Adv Neural Inf Process Syst 2022;35:12325–38.

[btae730-B8] Gimeno A , Ojeda-Montes MJ, Tomás-Hernández S et al The light and dark sides of virtual screening: what is there to know? Int J Mol Sci 2019;20:1375. 30893780 10.3390/ijms20061375 PMC6470506

[btae730-B9] Granville V , Krivánek M, Rasson J-P. Simulated annealing: a proof of convergence. IEEE Trans Pattern Anal Machine Intell 1994;16:652–6.

[btae730-B10] Guan J , Qian WW, Peng X et al 3D equivariant diffusion for target-aware molecule generation and affinity prediction. In: *The Eleventh International Conference on Learning Representations,* OpenReview.net, 2022.

[btae730-B11] Guan J , Zhou X, Yang Y et al DecompDiff: diffusion models with decomposed priors for structure-based drug design. In: *Proceedings of the 40th International Conference on Machine Learning,* pp. 11827–46. Round Rock, Texas, USA: PMLR, 2023.

[btae730-B12] Hao Y , Wang H, Liu X et al Deep simulated annealing for the discovery of novel dental anesthetics with local anesthesia and anti-inflammatory properties. Acta Pharm Sin B 2024;14:3086–109. 39027234 10.1016/j.apsb.2024.01.019 PMC11252475

[btae730-B13] Hu L , Benson ML, Smith RD et al Binding MOAD (mother of all databases). Proteins: Struct Funct Bioinform 2005;60:333–40. 10.1002/prot.20512 15971202

[btae730-B14] Huang Z , Yang L, Zhou X et al Protein–ligand interaction prior for binding-aware 3D molecule diffusion models. In: *The Twelfth International Conference on Learning Representations*. Kigali, Rwanda: OpenReview.net, 2023.

[btae730-B15] Huang Z , Yang L, Zhang Z et al Binding-adaptive diffusion models for structure-based drug design. AAAI 2024;38:12671–9.

[btae730-B16] Hwang C-R. Simulated annealing: theory and applications. PJM van Laarhoven and EHL Aarts: D. Reidel, Dordrecht. 1987. 198 pp., isbn 90-277-2513-6, dfl. 120, 1988.

[btae730-B17] Jensen JH. A graph-based genetic algorithm and generative model/Monte Carlo tree search for the exploration of chemical space. Chem Sci 2019;10:3567–72. 30996948 10.1039/c8sc05372c PMC6438151

[btae730-B18] Jin W , Barzilay R, Jaakkola T. Junction tree variational autoencoder for molecular graph generation. In: *Proceedings of the 35th International Conference on Machine Learning*, pp. 2323–32. PMLR, Stockholm, Sweden, 2018.

[btae730-B19] Jumper J , Evans R, Pritzel A et al Highly accurate protein structure prediction with alphafold. Nature 2021;596:583–9. 34265844 10.1038/s41586-021-03819-2 PMC8371605

[btae730-B20] Lipinski CA , Lombardo F, Dominy BW et al Experimental and computational approaches to estimate solubility and permeability in drug discovery and development settings. Adv Drug Deliv Rev 2012;64:4–17. 10.1016/s0169-409x(00)00129-0 11259830

[btae730-B21] Liu M , Luo Y, Uchino K et al Generating 3D molecules for target protein binding. In: *Proceedings of the 39th International Conference on Machine Learning,* pp.13912–24. Baltimore, Maryland, USA: PMLR, 2022.

[btae730-B22] Liu X , Li P, Meng F et al Simulated annealing for optimization of graphs and sequences. Neurocomputing 2021;465:310–24.

[btae730-B23] Liu X , Xue Z, Luo M et al Anesthetic drug discovery with computer-aided drug design and machine learning. APS 2024;2:7.

[btae730-B24] Long S , Zhou Y, Dai X et al Zero-shot 3d drug design by sketching and generating. Adv Neural Inf Process Syst 2022;35:23894–907.

[btae730-B25] Luo S , Guan J, Ma J et al A 3D generative model for structure-based drug design. In: *Proceedings of the 35th Conference on Neural Information Processing Systems,* Vol. 34. pp 6229–39. PMLR, 2021.

[btae730-B26] Meyers J , Fabian B, Brown N. De novo molecular design and generative models. Drug Discov Today 2021;26:2707–15. 34082136 10.1016/j.drudis.2021.05.019

[btae730-B27] Nigam A , Friederich P, Krenn M et al Augmenting genetic algorithms with deep neural networks for exploring the chemical space. In: *International Conference on Learning Representations*. OpenReview.net, 2020.

[btae730-B28] Peng X , Luo S, Guan J et al Pocket2mol: efficient molecular sampling based on 3d protein pockets. In: *International Conference on Machine Learning*, pp. 17644–55. Baltimore, Maryland, USA: PMLR, 2022.

[btae730-B29] Polishchuk PG , Madzhidov TI, Varnek A. Estimation of the size of drug-like chemical space based on gdb-17 data. J Comput Aided Mol Des 2013;27:675–9. 23963658 10.1007/s10822-013-9672-4

[btae730-B30] Qian H , Huang W, Tu S et al KGDIFF: towards explainable target-aware molecule generation with knowledge guidance. Brief Bioinform 2023;25:bbad435. 38040493 10.1093/bib/bbad435 PMC10783868

[btae730-B31] Qu Y , Qiu K, Song Y et al MolCRAFT: structure-based drug design in continuous parameter space. In: *Forty-first International Conference on Machine Learning*. Vancouver, Canada: PMLR, 2024.

[btae730-B32] Ragoza M , Masuda T, Koes DR. Generating 3D molecules conditional on receptor binding sites with deep generative models. Chem Sci 2022;13:2701–13. 35356675 10.1039/d1sc05976a PMC8890264

[btae730-B33] Schneuing A , Du Y, Harris C et al Structure-based drug design with equivariant diffusion models. arXiv, arXiv:2210.13695, 2022, preprint: not peer reviewed. 10.1038/s43588-024-00737-x PMC11659159 39653846

[btae730-B34] Stumpfe D , Hu H, Bajorath J. Evolving concept of activity cliffs. ACS Omega 2019;4:14360–8. 31528788 10.1021/acsomega.9b02221 PMC6740043

[btae730-B35] Wang T , Pulkkinen OI, Aittokallio T. Target-specific compound selectivity for multi-target drug discovery and repurposing. Front Pharmacol 2022;13:1003480. 36225560 10.3389/fphar.2022.1003480 PMC9549418

[btae730-B36] Xie Y , Shi C, Zhou H et al MARS: Markov molecular sampling for multi-objective drug discovery. In: *International Conference on Learning Representations*. OpenReview.net, 2021.

[btae730-B37] Xu K , Hu W, Leskovec J et al How powerful are graph neural networks? In: *International Conference on Learning Representations*. Vancouver, Canada: OpenReview.net, 2018.

[btae730-B38] Yang Y , Ouyang S, Hu X et al Structure-based drug design via 3D molecular generative pre-training and sampling. arXiv, arXiv:2402.14315, 2024, preprint: not peer reviewed.

[btae730-B39] Zhang Z , Liu Q. Learning subpocket prototypes for generalizable structure-based drug design. In: *International Conference on Machine Learning*, pp. 41382–98. Honolulu, Hawaii, USA: PMLR, 2023.

[btae730-B40] Zhang Z , Min Y, Zheng S et al Molecule generation for target protein binding with structural motifs. In: *The Eleventh International Conference on Learning Representations*. OpenReview.net, 2022.

[btae730-B41] Zhou X , Cheng X, Yang Y et al DecompOpt: controllable and decomposed diffusion models for structure-based molecular optimization. In: *The Thirteenth International Conference on Learning Representations*. Vienna, Austria: OpenReview.net, 2024.

[btae730-B42] Zhou Z , Kearnes S, Li L et al Optimization of molecules via deep reinforcement learning. Sci Rep 2019;9:10752. 31341196 10.1038/s41598-019-47148-x PMC6656766

[btae730-B43] Ziegler SJ , Mallinson SJ, John PCS et al Advances in integrative structural biology: towards understanding protein complexes in their cellular context. Comput Struct Biotechnol J 2021;19:214–25. 33425253 10.1016/j.csbj.2020.11.052 PMC7772369

