# Supplementary material for: TargetSA: adaptive simulated annealing for target-specific drug design
Source: Bioinformatics. 2024 Dec 4;41(1):btae730. doi: 10.1093/bioinformatics/btae730 (PMC12013812; doi:10.1093/bioinformatics/btae730)
Supplement: btae730_Supplementary_Data [file btae730_supplementary_data.pdf]

## More Details of Methodology

### Generation of Editing Frequency Matrix

Figure 1 shows how we obtain historical data from an editing-based molecule generation process, i.e., the molecule editing frequency matrix. Notably, when collecting historical data, editing positions are randomly selected.

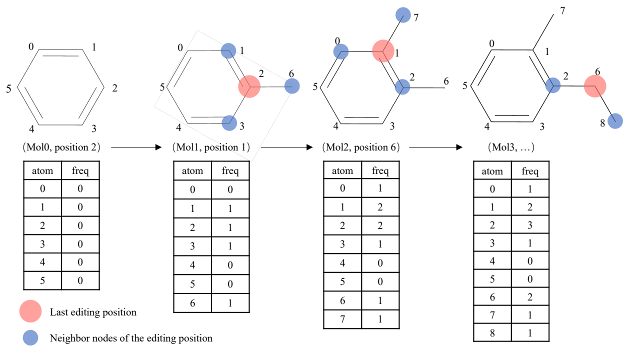


**Fig. 1.** Example of updating the editing frequency matrix during an editing-based molecular generation process.

### Training of the History-guided Position Predictor

Let $\mathrm{h}_{v_{r,i}}^{(k)}$ represent the feature vector of node $v_{r,i}$ at layer $k$, MLP be the multi-layer perceptions, $\mathcal{N}(v_{r,i})$ denotes the set of neighboring nodes of node $v_{r,i}$ in the graph, GIN updates node representations by

$$\mathrm{h}_{v_{r,i}}^{(k)} = \mathrm{MLP}^{(k)}\left(\left(1+\epsilon^{(k)}\right)\cdot \mathrm{h}_{v_{r,i}}^{(k-1)} + \sum\nolimits_{u\in\mathcal{N}(v_{r,i})} \mathrm{h}_{u}^{(k-1)}\right) \quad (1)$$

where $\epsilon$ is a learnable parameter used to scale the effect of self-loops, $\left(1+\epsilon^{(k)}\right)\cdot \mathrm{h}_{v_{r,i}}^{(k-1)}$ is the scaled sum of the node's own features. $\sum_{u\in\mathcal{N}(v_{r,i})} \mathrm{h}_{u}^{(k-1)}$ is the aggregated neighboring information obtained by summing the feature vectors of neighboring nodes $u$ in the previous layer $k-1$.

During the training process, let $\hat{\mathrm{q}_{\mathrm{r},\mathrm{i}}}$ be the predicted frequency of the atom $v_{r,i}$ and $\mathrm{q}_{r,i}$ is an associated frequency label. $\hat{Q_r}$ is the predicted frequency matrix of the molecule $\mathrm{x}_r$ and $Q_r$ is the ground truth.

$$\hat{\mathrm{q}_{\mathrm{r},\mathrm{i}}} = f_{\mathrm{freq}}(\mathrm{h}_{v_{r,i}}), \quad \hat{Q_r} = [\hat{\mathrm{q}_{\mathrm{r},0}}, \cdots, \hat{\mathrm{q}_{\mathrm{r},\mathrm{n}}}]^T \quad (2)$$

where $f_{\mathrm{freq}}$ is an atom frequency predictor using a series of fully connected layers and ReLU activation function. Cross-entropy loss $\mathcal{L}_{CE}$ is used to evaluate the difference in probability distributions between $Q_t$ and $\hat{Q_r}$, given by

$$\mathcal{L}_{CE}(Q_r, \hat{Q_r}) = -\sum_{i=1}^{m} \mathrm{q}_{\mathrm{r},\mathrm{i}} \log(\hat{\mathrm{q}_{\mathrm{r},\mathrm{i}}}) \quad (3)$$

Figure 2 illustrates how we utilize molecular graph features to forecast the editing frequency of each atom within a molecule, thereby identifying optimal positions for editing operations. For the settings of the graph neural network GIN: there is a total of five layers, and the embedding dimension is 300, only taking the embedding of the last layer's nodes as the output instead of summing all nodes. We have collected 49,897 historical data points, with a ratio of training: testing: validation = 8:1:1. Batch size is 128, the learning rate is 0.001, Adam optimizer is used, regularization parameter is 0.01, maximum training epochs are set to 500, and early stopping is based on validation set loss.

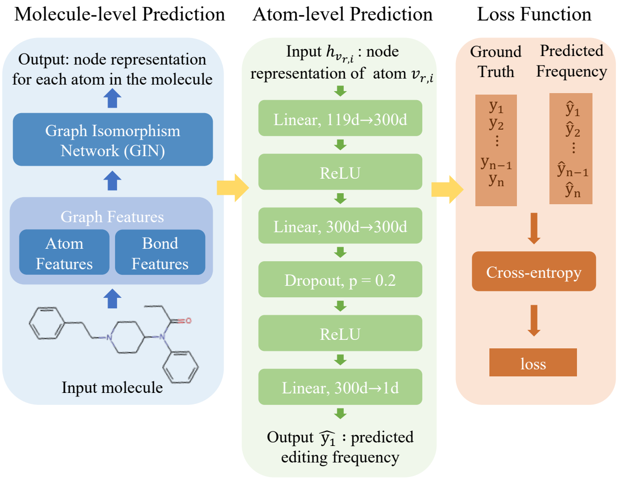


**Fig. 2.** Training procedures of the History-guided Position Predictor.

### Proof of Complete Operation Set

Let $\mathcal{T}$ denote a set of tree structures and $\mathcal{R}$ denote a set of ring structures, with $n$ and $m$ representing the number of elements in each set, respectively. Substructures from these sets can be linked by chemical bonds, forming a composite structure $\mathrm{x}_*$, as defined by

$$\mathrm{x}_* = \mathcal{T} \cup \mathcal{R}, \qquad \mathcal{T} = \{T_1, \cdots, T_n\}, \quad \mathcal{R} = \{R_1, \cdots, R_m\} \quad (4)$$

Any tree structure $T_*$ can be generated from a single atom $\mathrm{T}_0$ through editing operators from $\mathcal{A}_t$. Each insertion operator entails adding a new atom and linking it to an existing atom, while each replacement operator alters the atom type, thereby gradually building the tree structure without creating cycles. To generate a ring structure $R_*$, simply introduce a cyclization operator to the set $\mathcal{A}_r$ based on the tree generation process. We can close the loop of a tree structure $T_r$ (i.e. a linear chain of atoms) to form a ring or modify a ring structure $R_r$ to construct more complicated ring structures with different sizes and shapes.

$$T_* \in \mathcal{T}, \quad T_* = \mathrm{Gen}(T_0), \quad (5)$$
$$\mathrm{a}_t \sim \mathcal{A}_t, \quad \mathrm{A}_t = \{insertion, replacement\}$$
$$R_* \in \mathcal{R}, \quad R_* = \mathrm{Gen}(T_r) \ \textbf{or} \ R_* = \mathrm{Gen}(R_r), \quad (6)$$
$$\mathrm{a}_r \sim \mathcal{A}_r, \quad \mathrm{A}_r = \{insertion, replacement, cyclization\}$$

where $\mathrm{a}_t$ stands for the operator selected for generating $T_*$ and $\mathrm{a}_r$ for $R_*$. Since $\mathcal{A} = \mathcal{A}_r \cup \mathcal{A}_t$, it's safe to say that $\mathcal{A}$ is complete for molecule generation from scratch.

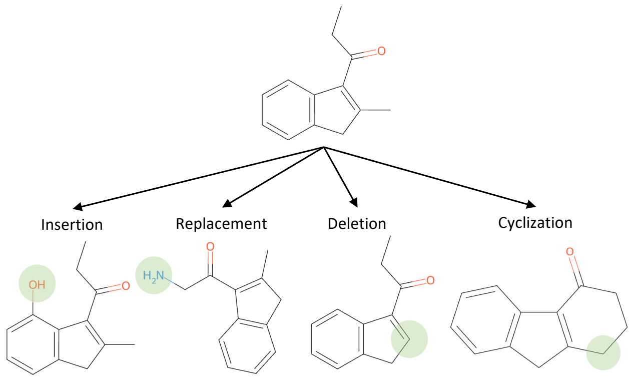


**Fig. 3.** Complete operation set with four operators: insertion, replacement, deletion, and cyclization.

# More Experimental Details

## Implementation Details

The hyperparameters utilized in the TargetSA framework are as follows: operation probabilities—(insertion: replacement: deletion: cyclization)—are 0.45, 0.2, 0.1, and 0.25, respectively. We determined these probabilities using 20 randomly selected pockets from the training set. The initial temperature $T_{init}$ is set to 1.0, and the final temperature $T_{min}$ to 0.1, with an inner loop of 5 iterations. Weights for properties in the objective function (Vina Dock: QED: SA) = (-3:2:3). The weights of each property and initial temperature $T_{init}$ were obtained through a grid search conducted on 20 randomly selected pockets. The experimental results are presented in Table 1, documenting the effect of different weights as well as the initial temperature of simulated annealing on the average docking scores. In our test experiments, we set the weight ratios to -3:2:3 as this configuration yielded the best results (Vina Dock -9.14). Setting $T_{init}$ to 1.0 is sufficient to obtain high-quality generation results. While higher $T_{init}$ values may yield better Vina Dock scores, they also significantly increase computational resource consumption and reduce efficiency.

Our method uses docking simulations to guide molecular optimization, which relies heavily on substantial CPU resources. Our server is equipped with 128 CPUs, specifically the AMD EPYC 7542 32-core Processor.

**Table 1.** Performances of TargetSA with different combinations of the initial temperature $T_{init}$ and the weights of properties in the objective function in terms of the docking score.

| Dock:QED:SA | $T_{init}$ | | | | |
|---|---|---|---|---|---|
| | 0.6 | 0.8 | 1.0 | 1.2 | 1.4 |
| -1:1:1 | -7.70 | -8.14 | -8.25 | -8.34 | -8.37 |
| -2:1:1 | -8.14 | -8.42 | -8.68 | -8.79 | -8.82 |
| -3:1:3 | -8.22 | -8.34 | -8.81 | -8.85 | -8.84 |
| -3:2:3 | -8.33 | -8.78 | -9.14 | -9.16 | -9.19 |

## Baselines

**LiGAN** (Ragoza et al. 2022) is a conditional VAE model that generates 3D ligands using atomic density grids from protein-ligand complexes; **AR** (Luo et al. 2021) autoregressively samples atoms with a high probability of occurrence until the binding site is filled. **Pocket2Mol** (Peng et al. 2022) leverages an E(3)-equivariant generative network to model features of the 3D pockets and proposes a more effective sampling approach. **GraphBP** (Liu et al. 2022) utilizes a normalizing flow framework and constructs local coordinate systems for precise atom type and location predictions. **FLAG** (Zhang et al. 2022) generates 3D molecules fragment-by-fragment. **DrugGPS** (Zhang and Liu 2023) innovates by modeling interactions between subpocket prototypes and fragments. **TargetDiff** (Guan et al. 2022) and **DecompDiff** (Guan et al. 2023) use diffusion-based SE(3)-equivariant networks to generate atom coordinates and types, with the latter extending to bond type generation. **IPDiff** (Huang et al. 2023) utilizes binding affinity signals for protein-ligand interaction learning, integrated into both diffusion and sampling processes. **DecompOpt** (Zhou et al. 2024) focuses on controllable diffusion for structure-based multi-objective optimization. **BindDM** (Huang et al. 2024) refines 3D molecule generation by adaptively extracting key elements of binding sites. **RGA** (Fu et al. 2022) is the most related work. It incorporates target structures as a modeling condition. It introduces an evolutionary Markov decision process, using molecular docking scores as an optimization objective for generating ligands bound tighter to targets.

## Fragment Library

Although our framework TargetSA can generate molecules from scratch, this methodology necessitates redundant optimization steps, rendering it highly time-consuming. Therefore, we collect a group of drug-like fragments as initial inputs. Each fragment undergoes an independent optimization process. The selection of initial drug-like fragments profoundly influences the outcome of the optimization process. We screened a group of 112 fragments deemed conducive to growth, sourced from a fragment library provided by Enamine, which is a global leader in drug discovery services with one of the world's largest collections of building blocks, fragments, and screening compounds (Carbery et al. 2022). The selection criteria are as follows:

1. Constituent elements limited to C, N, O, S, or halogens.
2. Total number of nitrogen and oxygen atoms is $>2$ and $<7$.
3. Number of ring structures ranging from 1 to 3.
4. Limits on halogen atom counts: fluorine atoms $<5$ , chlorine atoms $<3$, bromine atoms $<2$, sulfur atoms $<3$.
5. No more than 3 consecutive rotatable bonds.
6. Tanimoto similarity with other fragments $<0.8$.

# More Experimental Results

## Comparison with Previous Editing-based Methods

We compared TargetSA with previous editing-based methods such as RGA (Fu et al. 2022), MIMOSA (Fu et al. 2021), and MARS (Xie et al. 2021). Our approach differs in the following ways:

1. MARS and MIMOSA are only suitable for optimizing specific biological objectives (GSK3$\beta$ and JNK3), whereas TargetSA can also design novel molecules for any target.
2. Other methods lack the cyclization operation. Although MARS can add fragments with cycles, its generation quality is limited by the vocabulary of fragments. Our method can explore a broader drug-like space.
3. MARS considers only a single attachment position of fragments, while our history-guided position predictor

dynamically predicts the most suitable editing positions across the entire molecule.

4. RGA primarily generates molecules through crossover and mutation based on chemical reactions, differing from our step-by-step editing process. Additionally, RGA relies on extensive pre-training with ligand-protein complexes, which our method does not require.

For RGA, its average Vina Dock result is -8.30, while TargetSA achieves -9.09. TargetSA also outperforms it on the High Affinity metric by 14.8%. For MIMOSA and MARS, we have supplemented our work with additional multi-objective optimization experiments following their methodologies, optimizing PLogP + DRD and GSK3$\beta$ + JNK3 + QED + SA, respectively. As shown in Table 2, TargetSA achieves greater improvement in PLogP and DRD (0.81 and 0.47), compared to MIMOSA (0.75 and 0.35).

**Table 2.** Comparison of different models in terms of improvement in PLogP and DRD values, where DRD measures a molecule's biological activity against GSK3$\beta$ and JNK3.

| Method | PLogP-Imp. | DRD-Imp. |
|---|---|---|
| JT-VAE | 0.20 | 0.18 |
| VJTNN | 0.55 | 0.27 |
| GCPN | 0.38 | 0.25 |
| GA | 0.68 | 0.20 |
| MIMOSA | 0.75 | 0.35 |
| TargetSA | **0.81** | **0.47** |

The experiments concerning MARS are shown in Table 3, where PM is the product of the Success Rate, Novelty, and Diversity. TargetSA achieved the highest values in the baseline for three metrics Success Rate, Novelty, and PM (97.6%, 100.0%, 0.57), and Diversity is also maintained at a reasonable value.

**Table 3.** Comparison of different models in terms of Success Rate, Novelty, Diversity, and PM metrics.

| Method | Success Rate | Novelty | Diversity | PM |
|---|---|---|---|---|
| GCPN | 0.0% | 0.0% | 0.000 | 0.00 |
| JT-VAE | 5.4% | 100.0% | 0.277 | 0.02 |
| RationaleRL | 75.0% | 55.5% | 0.706 | 0.29 |
| GA+D | 85.7% | 100.0% | 0.363 | 0.31 |
| MARS | 92.3% | 82.4% | **0.719** | 0.55 |
| TargetSA | **97.6%** | **100.0%** | 0.624 | **0.57** |

## Performance on Complex Protein Structures

In real-world target-specific drug design, particularly challenging scenarios may arise involving complex targets. Proteins with extended amino acid sequences, intricate three-dimensional folding patterns, or multiple functional domains present significant difficulties in drug design. Therefore, we analyzed TargetSA on 10 targets with the longest amino acid sequences from the Crossdocked 2020 test set. The results are detailed in Table 4. It can be observed that TargetSA consistently delivers superior generation results for complex target proteins. This underscores the effectiveness of our editing-based molecular generation strategy, compared with existing popular deep generative models.

**Table 4.** Generation results on 10 complex target proteins.

| Model \ Metric | Vina Dock (↓) | High Affinity (↑) | QED (↑) | SA (↑) |
|---|---|---|---|---|
| AR | -7.04 | 35.6% | 0.45 | 0.64 |
| Pocket2Mol | -7.87 | 62.2% | 0.56 | **0.76** |
| TargetDiff | -8.54 | 68.2% | 0.43 | 0.56 |
| TargetSA | **-9.84** | **81.6%** | **0.57** | 0.67 |
| Reference | -7.70 | - | 0.37 | 0.71 |

## More Evaluation Metrics for 2D Molecular Graphs

Based on previous methods, we have introduced four additional evaluation metrics: ring size, number of rotatable bonds (**ROT**), number of hydrogen bond acceptors (**HBA**), and number of hydrogen bond donors (**HBD**). The results are presented in table 5 and figure 4.

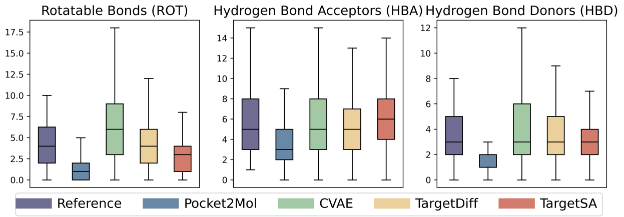


**Fig. 4.** Evaluation of ROT, HBA and HBD for reference and generation results of different models.

**Table 5.** Evaluation of ring sizes for reference and generation results of different models, where "Ref" means reference and "P2M" means Pocket2Mol.

| Ring Size | Ref. | LiGAN | AR | P2M | TargetDiff | Ours |
|---|---|---|---|---|---|---|
| 3 | 1.7% | 28.1% | 29.9% | 0.1% | 0.0% | 0.6% |
| 4 | 0.0% | 15.7% | 0.0% | 0.0% | 2.8% | 0.0% |
| 5 | 30.2% | 29.8% | 16.0% | 16.4% | 30.8% | 27.8% |
| 6 | 67.4% | 22.7% | 51.2% | 80.4% | 50.7% | 71.5% |
| 7 | 0.7% | 2.6% | 1.7% | 2.6% | 12.1% | 0.1% |
| 8 | 0.0% | 0.8% | 0.7% | 0.3% | 2.7% | 0.0% |
| 9 | 0.0% | 0.3% | 0.5% | 0.1% | 0.9% | 0.0% |

## Evaluation the Novelty of Generated Ligands

We further evaluated the novelty, similarity, uniqueness, and diversity of the generated molecules, as presented in Table 6. **Novelty** indicates the proportion of generated molecules within the same pocket that differ from the reference. **Similarity** represents the average Tanimoto similarity of the generated ligands compared to the reference. **Uniqueness** refers to the proportion of unique molecules among all generated ones. **Diversity** retains its previous definition.

The results demonstrate that TargetSA ensures 100% novelty and uniqueness while maintaining low similarity to the reference molecule. Our method is capable of generating novel compounds, which is critical for novel drug discovery.

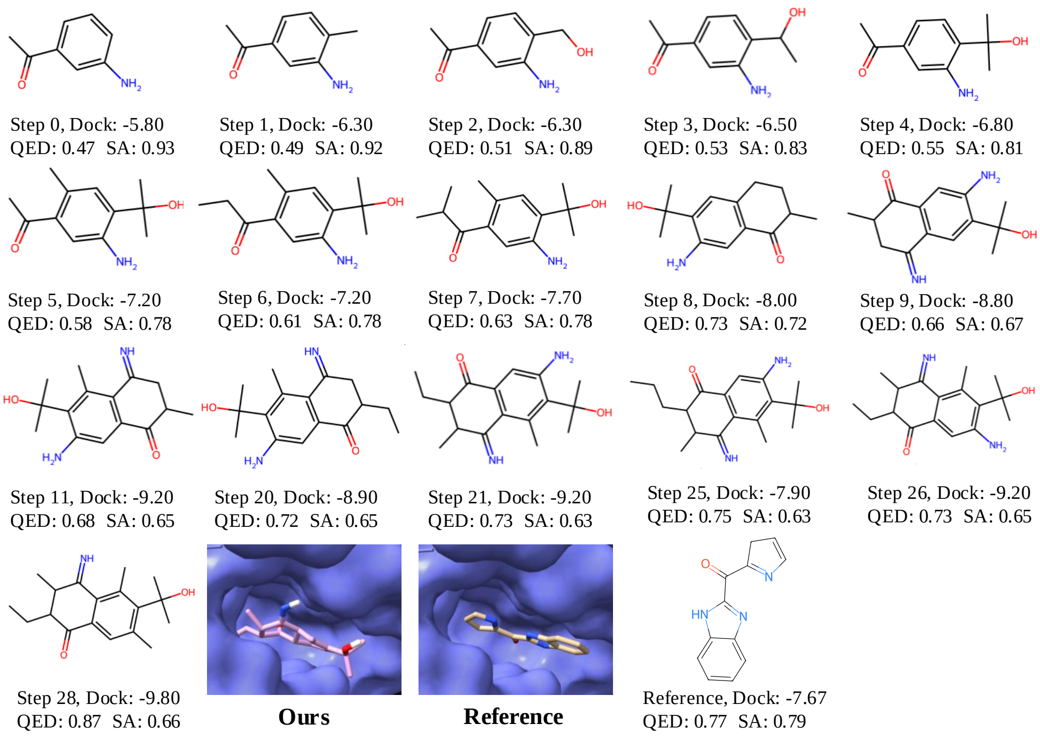


**Fig. 5.** Examples of a complete molecular editing process of using TargetSA to generate novel compounds.

**Table 6.** Evaluation of the ability to design novel ligands.

| Methods | Novelty | Similarity | Uniqueness | Diversity |
|---|---|---|---|---|
| LiGAN | 100% | 0.22 | 87.82% | 0.66 |
| AR | 100% | 0.24 | 100% | 0.70 |
| Pocket2Mol | 100% | 0.26 | 100% | 0.69 |
| TargetDiff | 100% | 0.30 | 99.63% | 0.72 |
| DecompDiff | 100% | 0.34 | 99.99% | 0.68 |
| RGA | 100% | 0.37 | 96.82% | 0.41 |
| DecompOpt | 100% | 0.36 | 100% | 0.60 |
| TargetSA | 100% | 0.26 | 100% | 0.59 |

## Case Study of A Complete Molecular Generation and Optimization Process

As shown in Figure 5, after a complete optimization process, the final generated molecule exhibited significantly enhanced binding affinity with a specific target (PDB id: 4AUA). Also, the QED value is notably improved and SA is maintained in an acceptable range.

## Evaluation of Generating Time

The average time for TargetSA to generate 100 molecules for each pocket is 12.7 hours, and a comparison with other models is shown in Table 7. Although AR's generation time was a third of ours, its generation result (Vina Dock -6.75) was substantially worse than TargetSA's (-9.09). We acknowledge that our TargetSA sacrifices some computational efficiency to achieve better generation results, such as higher binding affinity. This trade-off arises from two main factors. First, docking simulations are inherently resource-intensive due to their precision and dynamic nature. Despite this, docking tools remain among the most crucial instruments in the field of drug discovery. Second, combinatorial optimization methods (e.g., simulated annealing), which directly search through the discrete chemical space, exhibit a trade-off between computation time and the extent of chemical space exploration. In contrast, deep generative models, which rapidly generate molecules by imitating molecular data distributions, typically require extensive data for training. This data requirement limits their effectiveness in low-data scenarios. Our TargetSA avoids the need for large amounts of protein-ligand pair data for pre-training, thereby conserving computational resources.

Admittedly, our method is relatively time-consuming, but it doesn't limit scalability. Given that real-world drug development often spans decades, our computational time is relatively modest. Additionally, runtime is not the most critical metric in target-specific drug design. The primary goal is to generate novel compounds with tighter binding to the target, rather than producing a large volume of molecules in a short time. Our TargetSA achieves SOTA performance on the Vina Dock metric, demonstrating its promising application potential.

**Table 7.** Average time of generating 100 molecules for each pocket and the time metric is in hours.

| Metric / Model | Vina Dock (↓) | High Affinity (↑) | Time (↓) |
|---|---|---|---|
| LiGAN | -6.33 | 21.1% | <1 |
| GraphBP | -4.80 | 14.2% | <1 |
| AR | -6.75 | 37.9% | 4.5 |
| Pocket2Mol | -7.15 | 48.4% | <1 |
| FLAG | -6.96 | 44.5% | <1 |
| DrugGPS | -7.27 | 56.5% | <1 |
| TargetSA | -9.09 | 79.4% | 12.7 |

## Examples of Generated Molecules with Lower Affinities Than the Reference

Here we provide examples where the generated molecules exhibit lower affinities than the reference, as shown in figure 6. These molecules are more likely to fail in binding to the target pocket (PDB ID: 3TYM). The possible reasons for these failures are as follows:

1. The complexity of pocket structure: Even advanced docking simulation tools may struggle to accurately model highly complex pockets, limiting their effectiveness in drug discovery.
2. Deletion operations in our molecule editing process: TargetSA has a small probability of deleting substructures at the later stages of optimization, resulting in final molecules that only partially occupy the pocket.
3. Pattern differences between generated molecules and reference molecules: For example, our generated examples form concentrated ring structures, while the reference molecule has more dispersed rings.

In the future, we aim to enhance our method to better utilize the precise 3D structural information of pockets, enabling the generation of effective molecules to tackle the aforementioned issues.

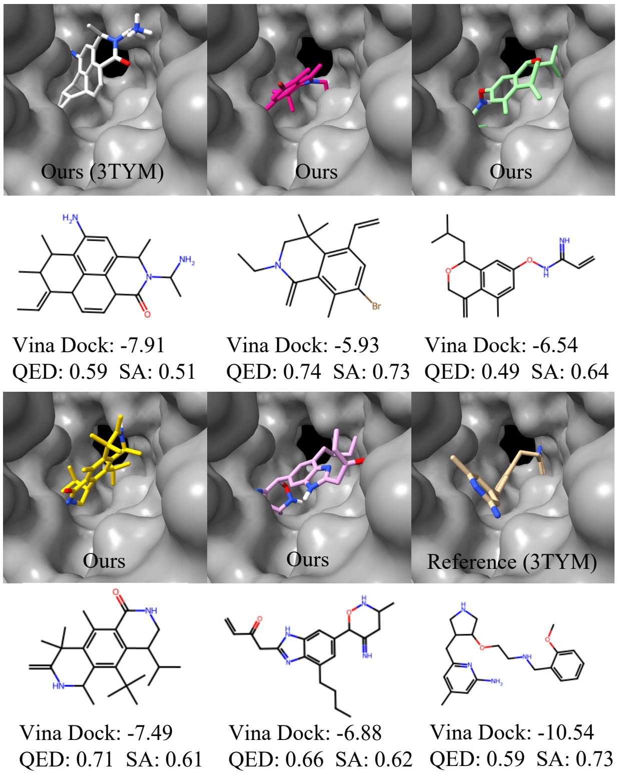


**Fig. 6.** Examples of a specific pocket (PDB ID: 3TYM): generated molecules demonstrating lower affinity than the reference ligand.

## Docking Results of Drug-like Fragments

The docking results of drug-like fragments are presented in the table 8. Analysis reveals that the initial docking scores of drug-like fragments notably lag behind those of reference molecules. In other words, we selected a drug-like fragment library primarily to accelerate the optimization process and avoid the computationally intensive of generating ligands from scratch, rather than initially selecting fragments with high docking scores. It's clear that, after the editing operations by TargetSA, the docking affinity of the resultant generated molecules exhibits a marked enhancement, demonstrating the effectiveness of our approach.

**Table 8.** Docking results of drug-like fragments and reference, where "First Dock" denotes the average docking scores between drug-like fragments and targets, "Final Dock" stands for the docking score of the final generated molecules optimizing from these fragments, and "Dock Improvement" is the difference between the two.

| Metric \ Dataset | Crossdocked 2020 | Binding MOAD |
|---|---|---|
| Reference | -7.45 | -6.83 |
| First Dock | -5.45 | -5.27 |
| Final Dock (↓) | -9.09 | -7.95 |
| Dock Improvement (↑) | 3.64 | 2.68 |

## References

A. Carbery, R. Skyner, F. von Delft, and C. M. Deane. Fragment libraries designed to be functionally diverse recover protein binding information more efficiently than standard structurally diverse libraries. *Journal of Medicinal Chemistry*, 65(16):11404–11413, 2022.

T. Fu, C. Xiao, X. Li, L. M. Glass, and J. Sun. Mimosa: Multi-constraint molecule sampling for molecule optimization. In *Proceedings of the AAAI Conference on Artificial Intelligence*, volume 35, pages 125–133, 2021.

T. Fu, W. Gao, C. Coley, and J. Sun. Reinforced genetic algorithm for structure-based drug design. *Advances in Neural Information Processing Systems*, 35:12325–12338, 2022.

J. Guan, W. W. Qian, X. Peng, Y. Su, J. Peng, and J. Ma. 3d equivariant diffusion for target-aware molecule generation and affinity prediction. In *The Eleventh International Conference on Learning Representations*, 2022.

J. Guan, X. Zhou, Y. Yang, Y. Bao, J. Peng, J. Ma, Q. Liu, L. Wang, and Q. Gu. Decompdiff: Diffusion models with decomposed priors for structure-based drug design. In *International Conference on Machine Learning*, pages 11827–11846. PMLR, 2023.

Z. Huang, L. Yang, X. Zhou, Z. Zhang, W. Zhang, X. Zheng, J. Chen, Y. Wang, C. Bin, and W. Yang. Protein-ligand interaction prior for binding-aware 3d molecule diffusion models. In *The Twelfth International Conference on Learning Representations*, 2023.

Z. Huang, L. Yang, Z. Zhang, X. Zhou, Y. Bao, X. Zheng, Y. Yang, Y. Wang, and W. Yang. Binding-adaptive diffusion models for structure-based drug design. *Proceedings of the AAAI Conference on Artificial Intelligence*, 38:12671–12679, Mar. 2024. doi: 10.1609/aaai.v38i11.29162.

M. Liu, Y. Luo, K. Uchino, K. Maruhashi, and S. Ji. Generating 3d molecules for target protein binding. In *International Conference on Machine Learning*, pages 13912–13924. PMLR, 2022.

S. Luo, J. Guan, J. Ma, and J. Peng. A 3d generative model for structure-based drug design. In M. Ranzato, A. Beygelzimer, Y. Dauphin, P. Liang, and J. W. Vaughan, editors, *Advances in Neural Information Processing Systems*, volume 34, pages 6229–6239. Curran Associates, Inc., 2021.

X. Peng, S. Luo, J. Guan, Q. Xie, J. Peng, and J. Ma. Pocket2mol: Efficient molecular sampling based on 3d protein pockets. In *International Conference on Machine Learning*, pages 17644–17655. PMLR, 2022.

M. Ragoza, T. Masuda, and D. R. Koes. Generating 3d molecules conditional on receptor binding sites with deep generative models. *Chemical science*, 13(9):2701–2713, 2022.

Y. Xie, C. Shi, H. Zhou, Y. Yang, W. Zhang, Y. Yu, and L. Li. Mars: Markov molecular sampling for multi-objective drug discovery. In *International Conference on Learning Representations*, 2021.

Z. Zhang and Q. Liu. Learning subpocket prototypes for generalizable structure-based drug design. In *International Conference on Machine Learning*, pages 41382–41398. PMLR, 2023.

Z. Zhang, Y. Min, S. Zheng, and Q. Liu. Molecule generation for target protein binding with structural motifs. In *The Eleventh International Conference on Learning Representations*, 2022.

X. Zhou, X. Cheng, Y. Yang, Y. Bao, L. Wang, and Q. Gu. Decompopt: Controllable and decomposed diffusion models for structure-based molecular optimization. In *The Thirteenth International Conference on Learning Representations*, 2024.
